# Rapamycin regulates autophagy and cell adhesion in induced pluripotent stem cells

**DOI:** 10.1186/s13287-016-0425-x

**Published:** 2016-11-15

**Authors:** Areechun Sotthibundhu, Katya McDonagh, Alexander von Kriegsheim, Amaya Garcia-Munoz, Agnieszka Klawiter, Kerry Thompson, Kapil Dev Chauhan, Janusz Krawczyk, Veronica McInerney, Peter Dockery, Michael J. Devine, Tilo Kunath, Frank Barry, Timothy O’Brien, Sanbing Shen

**Affiliations:** 1 Regenerative Medicine Institute, School of Medicine, National University of Ireland Galway, Galway, Ireland; 2 Systems Biology Ireland, Conway Institute, University College Dublin, Dublin 4, Ireland; 3 Centre for Microscopy and Imaging, Anatomy, School of Medicine, National University of Ireland Galway, Galway, Ireland; 4 Department of Haematology, Galway University Hospital, Galway, Ireland; 5 HRB Clinical Research Facility, National University of Ireland Galway, University Road, Galway, Ireland; 6 MRC Center for Regenerative Medicine, The University of Edinburgh, Edinburgh, UK; 7 Department of Molecular Neuroscience, Institute of Neurology, University College London, London, WC1N 3BG UK; 8 Chulabhorn International College of Medicine, Thammasat University, Patumthani, 12120 Thailand

**Keywords:** Actin cytoskeleton, Adherens junctions, Autophagy, Differentiation, Embryoid body, Induced pluripotent stem cells, Rapamycin

## Abstract

**Background:**

Cellular reprogramming is a stressful process, which requires cells to engulf somatic features and produce and maintain stemness machineries. Autophagy is a process to degrade unwanted proteins and is required for the derivation of induced pluripotent stem cells (iPSCs). However, the role of autophagy during iPSC maintenance remains undefined.

**Methods:**

Human iPSCs were investigated by microscopy, immunofluorescence, and immunoblotting to detect autophagy machinery. Cells were treated with rapamycin to activate autophagy and with bafilomycin to block autophagy during iPSC maintenance. High concentrations of rapamycin treatment unexpectedly resulted in spontaneous formation of round floating spheres of uniform size, which were analyzed for differentiation into three germ layers. Mass spectrometry was deployed to reveal altered protein expression and pathways associated with rapamycin treatment.

**Results:**

We demonstrate that human iPSCs express high basal levels of autophagy, including key components of APMKα, ULK1/2, BECLIN-1, ATG13, ATG101, ATG12, ATG3, ATG5, and LC3B. Block of autophagy by bafilomycin induces iPSC death and rapamycin attenuates the bafilomycin effect. Rapamycin treatment upregulates autophagy in iPSCs in a dose/time-dependent manner. High concentration of rapamycin reduces NANOG expression and induces spontaneous formation of round and uniformly sized embryoid bodies (EBs) with accelerated differentiation into three germ layers. Mass spectrometry analysis identifies actin cytoskeleton and adherens junctions as the major targets of rapamycin in mediating iPSC detachment and differentiation.

**Conclusions:**

High levels of basal autophagy activity are present during iPSC derivation and maintenance. Rapamycin alters expression of actin cytoskeleton and adherens junctions, induces uniform EB formation, and accelerates differentiation. IPSCs are sensitive to enzyme dissociation and require a lengthy differentiation time. The shape and size of EBs also play a role in the heterogeneity of end cell products. This research therefore highlights the potential of rapamycin in producing uniform EBs and in shortening iPSC differentiation duration.

**Electronic supplementary material:**

The online version of this article (doi:10.1186/s13287-016-0425-x) contains supplementary material, which is available to authorized users.

## Background

Induced pluripotent stem cell (iPSC) technology enables conversion of patients’ somatic cells into embryonic stem (ES)-like cells [1] which can be differentiated into the major cell types in the body, raising the expectation of personalized medicine to treat patients with their own somatic tissue-derived cells. This technology also offers an opportunity to generate human cell models of diseases for therapeutic development, which has not kept pace with pharmaceutical investment in recent decades. A major impediment to drug discovery is attributable to the lack of suitable disease models of human origin, because species differences may totally affect drug efficacy [2]. Some disease pathologies are shown to be recapitulated in the culture dish. For example, many patient-specific iPSCs have been generated to investigate disease progression in vitro and disease-related phenotypes are partially preserved in iPSC-derived cells [3–6]. Recent successes in the growth of “mini-organs”—that is, 3D retina [7, 8] and cerebral organoids [9]—may provide 3D human disease models. However, there are challenges in phenotyping iPSCs prior to the development of screening assays, which include lengthy differentiation durations and heterogeneity of the end cell products.

The differentiation, survival, and self-renewal of human stem cells can be regulated by a 3D microenvironment including shape and size [7–10]. Human iPSCs are hypersensitive to cell dissociation reagents, and seconds of exposure to Trypsin/EDTA may lead to failure of iPSC adhesion or survival. Consequently, many laboratories adopt a “cut-and-paste” method to mechanically passage iPSCs, which results in embryoid bodies (EBs) of uncontrollable sizes with irregular shapes, which may potentially contribute to the heterogeneity. The differentiation of mature cell types from human iPSCs is also time consuming. For example, 3–5 months are required to generate functional neurons, astrocytes, or oligodendrocytes [11–13]. Therefore, accelerated differentiation remains to be optimized.

Efforts have been made to improve the reprogramming efficiency, and autophagy, a process to degrade unwanted proteins, is found to be one of the regulators during cellular reprogramming [14, 15]. Autophagy is conserved from yeast to mammals, and is generally induced by intracellular and extracellular stress. Upon induction, the preautophagosomal structure is formed and elongated to form a phagophore, which engulfs cytoplasmic components, leading to the formation of autophagosomes with double membranes. The genes involved in the autophagy are termed autophagy-related genes (*ATG*s), and >12 *ATGs* have been identified. They regulate autophagosome formation through two evolutionarily conserved ubiquitin-like conjugation systems, the ATG12–ATG5 and the ATG8 (LC3)–PE (phosphatidylethanolamine) systems [16]. Microtubule-associated proteins 1A/1B light chain 3-I (LC3B-I) is conjugated with PE to become LC3B-II, which associates with both the outer and inner membranes of the autophagosome. After fusion with the lysosome, the autolysosome is degraded [17]. In mice, Atg3, Atg5, and Atg7 are essential for reprogramming of mouse embryonic fibroblasts [14, 15]. Cells lacking Atg3, Atg5, or Atg7 abrogate iPSC colony formation [15].

The autophagy pathway can be activated by AMPK signaling, but is normally inhibited by the mammalian target of rapamycin (mTOR) pathway. The presence of hyperactivated mTOR activity in *Tsc2* ^*–*/–^ somatic cells completely blocked reprogramming [14]. Consistent with this, rapamycin (an inhibitor of the mTOR pathway at 0.3–1 nM) could increase reprogramming efficiency by 2–3-fold [18]. In addition, 0.1 nM of PP242, a selective inhibitor of mTORC1/mTORC2 binding at the ATP domain, also increased reprogramming efficiency by 5-fold [18]. Other compounds associated with autophagy such as PQ401 (an IGF1 receptor inhibitor) and LY294002 (an inhibitor of PI3K) also increased reprogramming by 4-fold [18]. Mechanistically, mTOR is regulated by SOX2, one of the four reprogramming factors. SOX2 can physically bind to the mTOR promoter and repress mTOR expression, thereby activating autophagy [15]. An appropriate level of autophagy is therefore required for cell reprogramming. However, roles of autophagy in iPSC maintenance and differentiation remain elusive.

In this study, we showed the presence of high levels of basal autophagy activity during iPSC reprogramming and maintenance. Rapamycin alters expression of adherens junctions and actin cytoskeleton, induces iPSC detachment, and results in uniform EB formation. Rapamycin treatment also accelerates differentiation of human iPSCs into three germ layers.

## Methods

### iPSC derivation

On day 0, human fibroblasts were seeded at a density of 2.0 × 10^4^ cells/well on a six-well dish. Next day, cells were transduced with a polycistronic (OKSM) lentivirus containing four reprogramming factors (SCR544-Human STEMCCA constitutive lentivirus reprogramming kit, SCR544; Millipore) in 700 μl of fibroblast medium, supplemented with 4 μg/ml polybrene (TR-1003; Millipore). On day 2, cells were repeatedly transduced with lentivirus. The medium was then replaced daily with complete fibroblast medium supplemented with 0.25 mM sodium butyrate (NaB, 156-54-7; Sigma) for 4 days. On day 6, six-well dishes were preseeded with γ-irradiated MEF (07GSC6001G, VH Bio Limited) at a density of 1.5 × 10^5^ cells/well. On day 7, transduced cells were trypsinized with 0.25 % Trypsin/EDTA, replated onto preseeded MEF dishes at a density of 3.0 × 10^4^ cells/well, and cultured in KO iPSC medium supplemented with 10 ng/ml bFGF (100-18B; Peprotech) plus 0.25 mM NaB. The medium was changed every other day. The addition of NaB was discontinued after day 20.

Individual iPSC colonies began to emerge after day 14 and were picked up on day 28, mechanically dissociated into smaller pieces by pipetting, and plated into γ-MEF-coated 12-well plates. Selected clones were characterized using alkaline phosphatase activity, RT-PCR, and immunoblotting to detect endogenous expression of OCT4, SOX2, and NANOG, and immunocytochemistry to detect surface markers of SSEA4, TRA 1-60, and TRA 1-81 in addition to the transcription factors (StemLight™ pluripotency antibody kit, 9656; Cell Signaling Technology).

### Cell culture

Human iPSC lines were maintained on a feeder-free culture system. The six-well plates were precoated with Geltrex, LDEV-free hESC-qualified reduced growth factor basement membrane matrix (A1413302; Invitrogen). Geltrex was thawed overnight at 4 °C and diluted 1:100 in KO-DMEM medium (10829018; Invitrogen). Each well was coated with 1.5 ml Geltrex and incubated for 1 hour at 37 °C, which was then replaced with the mTeSR1 medium (05850; StemCell Technologies) with 10 ng/ml bFGF at 37 °C and 5 % CO_2_. iPSCs were cut and pasted into Geltrex-coated wells. The medium was renewed daily and passaged every 6 days. Chemicals such as rapamycin were then added to respective experiments.

For induction of autophagy signaling, iPSC colonies were cultured in mTeSR1 medium supplemented with 0–300 nM rapamycin (R8781; Sigma) for 1–9 days. For autophagic flux assay, human iPSCs were treated with 200 nM rapamycin in the absence or presence of 50 nM bafilomycin A1 for 24 hours (B1793; Sigma) as specified. Cells were collected and protein was subsequently analyzed by immunoblotting.

### EB formation by cut-and-paste method

The cut-and-paste method was deployed to make conventional EBs as iPSCs. Spontaneously differentiated cells were removed under a stereomicroscope inside the culture safety cabinet, and fresh medium was added to the remaining iPSC colonies, which were cut into small pieces with a 10 μl pipette tip. The iPSC sections were then scraped off the dish and transferred to a T25 flask in 15 ml EB medium (KO DMEM, KO serum, 1× l-glut, and 1× NNEA plus 50 μl β-mercaptoethanol). The medium was changed every other day, and the EBs were cultured for 7–10 days before plating out for spontaneous differentiation assays.

### Antibodies and reagents

The reagents for transmission electron microscopy (TEM) include sodium cacodylate (C0250; Sigma), low viscosity resin kit (AGR1078A; Agar Scientific), 2 % osmium tetroxide (AGR1016; Agar Scientific), 25 % glutaraldehyde solution (23114.02; AMSBIO), and thermanox plastic coverslips (NUNC). For immunocytochemistry, the following antibodies were used: anti-LAMP1 (25630), anti-Syntaxin-6 (12370), and anti-NESTIN (105389) from Abcam; SelectFX® Alexa Fluor® 488 Endoplasmic Reticulum labeling kit (S34200) from Life Technologies; and anti-LC3B from autophagy antibody sampler kit (4445), Alexa Fluor® 488 Goat Anti-Rabbit IgG fluorescent dye (4412), and Alexa Fluor® 555 Goat Anti-Mouse IgG fluorescent dye (4409) from Cell Signaling Technology. For immunoblot experiments, reagents included: anti-α-fetoprotein (A8452), anti-α-smooth muscle actin (A2547), anti-ATG13 (SAB3500502), and ATG101 (SAB3500503) from Sigma; anti-βIII tubulin (G7121) from Promega Corporation; anti-ULK2 (56736) from Abcam; and anti-LC3B, anti-BECLIN-1, anti-ATG5 (D1G9; #8540P, 1:1000), anti-ATG12 and anti-ATG3 autophagy antibody sampler kit (4445), anti-p-ULK1 (Ser317, 6887), anti p-p70S6K (Thr389, 9205), anti-β-Actin (4967), HRP-conjugated anti-mouse IgG antibody (7076), and HRP-conjugated anti-rabbit-IgG antibody (7074) from Cell Signaling Technology. MitoGreen (a green-fluorescent mitochondrial dye) was purchased from Promokine (PK-CA707-70054).

### Transmission electron microscopy

iPSCs were cultured on Thermanox coverslips in six-well plates coated with Geltrex, rinsed with prewarmed (37 °C) 0.1 M cacodylate buffer, fixed with 2 % glutaraldehyde and 2 % paraformaldehyde in 0.1 M cacodylate buffer for 3 hours at room temperature, rinsed again with cacodylate buffer, and post fixed with 1 % osmium tetroxide in cacodylate for 1 hour at room temperature. Samples were then rinsed with cacodylate buffer, dehydrated through a series of graded ethanol, embedded in low-viscosity resin according to the standard protocol, and polymerized at 60 °C for 48 hours. Ultra-thin sections were obtained using a diamond knife, on a Leica Reichert Jung ultra-microtome, and stained with the contrasting agents, uranyl acetate and lead citrate, in a Leica EM AC20 stainer. Sections were examined with a Hitachi H7000 transmission electron microscope fitted with a 1 K Hamamatsu digital camera, and images were captured using AMTV542 Image Capture Engine software.

### Immunocytochemistry

To investigate the effect of rapamycin, iPSC colonies or the EB-like spheres were transferred to a μ-Slide eight-well glass bottom flask (80826; ibidi GmBH) coated with Geltrex. Cells were fixed with 4 % paraformaldehyde for 10 min at room temperature and washed with PBS three times before permeabilization using 0.5 % Triton X-100 for 10 min. Nonspecific binding was blocked with 5 % normal goat serum in PBS containing 0.5 % Trion X for 1 hour prior to incubation with primary antibodies overnight at 4 °C. The wells were washed three times with PBS and incubated with secondary antibodies for 1 hour at room temperature. The nuclei were visualized by Hoechst counterstaining for 10 min. After washing with PBS, the immunofluorescence was visualized with the Andor Revolution spinning disk confocal microscope, using Andor IQ2 software. The samples were imaged using a combination of λ405 nm, λ488 nm and λ564 nm lasers.

### Immunoblotting

Following experimental treatments, cell pellets were resuspended in NP40 cell lysis buffer (FNN0021; Invitrogen) supplemented with protease inhibitor cocktail (P2714; Sigma). Lysate was then centrifuged at 13,000 rpm for 10 min at 4 °C, and the supernatant was collected and used for immunoblotting. The protein concentration was determined and the samples were denatured in sample buffer (62.5 mM Tris–HCl pH 6.8, 2 % SDS, 10 % glycerol, 2 % mercaptoethanol, and 0.01 % bromophenol blue) at 95 °C for 5 min. Proteins were resolved on 10–15 % SDS-PAGE gels and then electrophoretically transferred to a polyvinylidene difluoride (PVDF) membrane (162-0177; Bio-Rad). The transfer efficiency was checked by Ponceau-S red staining (P7170; Sigma). The membranes were washed with Tris-buffered saline containing 0.1 % Tween-20 (TBST) for 5 min, incubated in blocking buffer for 1 hour at room temperature, and incubated overnight at 4 °C with primary antibodies. They were then washed three times for 5 min each with TBST, incubated with secondary antibodies for 1 hour at room temperature, and washed in TBST. Finally, the membranes were visualized by enhanced chemiluminescence using ECL™ Prime (GZ28980926; Amersham Biosciences). The immunoblot band densities were normalized with the unphosphorylated protein, actin, or GAPDH, and quantified using a densitometer in the Scion image analysis program (National Institutes of Health, Bethesda, MD, USA).

### Data analysis and statistical methods

Data were expressed as the mean ± standard error of means (SEM) or mean ± SD as indicated. Significance was assessed using one-way analysis of variance (ANOVA) followed by the Tukey–Kramer test using GraphPad Prism version 5. *p* < 0.05 was considered significant, and *p* < 0.01 very significant.

### Mass spectrometry analysis

The iPSCs were lysed in 1 % SDS and the lysates were processed using the FASP protocol as published previously [19]. Briefly, the cell lysates were sonicated, detergents were removed by sequential washes in spin columns, and proteins were digested with trypsin. The peptides were analyzed on a Q-Exactive Mass Spectrometer as described previously [20]. The proteins were identified and quantified with the MaxQuant 1.5 software suite by searching against the human uniprot database, with N-terminal actylation and methionine oxidation as variable modification and a FDR of 0.01. Mass accuracy was set a 4.5 ppm for the MS and 20 ppm for the MS/MS. LFQ was performed by MaxLFQ as described previously [21]. One-way ANOVA was carried out to compare rapamycin-treated samples with control iPSCs. The mean expression levels (M) and SEM were quantified, and folds of changes in the treated samples were calculated against the control iPSCs (set as 1). *p* < 0.05 was considered statistically significant. A total of 220 proteins which were significantly altered after rapamycin treatment were analyzed by the STRING program for pathway using GO biological processes and GO cellular components.

## Results

### Autophagy is a prominent feature of reprogramming cells and stable iPSCs

**Fig. 1:**
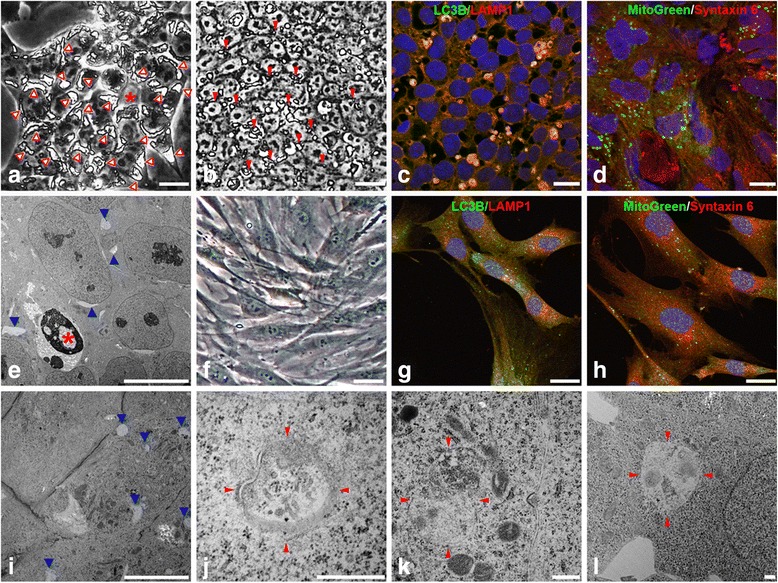
Autophagy machinery is widely operated in iPSCs. (**a**) Images of cells taken at day 15 of fibroblast reprogramming and large autophagic vacuoles indicated (*red arrowheads*). (**b**) Smaller autophagic vacuoles (*red arrowheads*) were observed in stable iPSC lines with daily change of culture medium. (**c**, **d**, **g**, **h**) Double immunofluorescence staining of iPSCs (**c**, **d**) and fibroblasts (**g**, **h**) was carried out with anti-LC3B for autophagy (**c**, **g** *green*) and anti-LAMP1 for lysosome (**c**, **g** *red*), Syntaxin 6 for Golgi membrane (**d**, **h** *red*) and MitoGreen (**d**, **h** *green*) for mitochondria, with counter-staining of DAPI (*blue*) for nuclei. Fluorescent images were acquired via confocal microscopy. Note that LC3B and LAMP1 are colocalized in iPSCs (**c**) but not in fibroblasts (**g**), whereas Syntaxin 6/MitoGreen are not colocalized as anticipated (**d**, **h**). (**e**, **i**–**l**) TEM images showed a dead nucleus (* **a**, **e**), lysosomal structures (*blue arrowheads*, **e**, **i**), and autophagic vacuoles (*red arrowheads*, **j**–**l**). *Bar* = 20 μm **a**–**d** and **f**–**h**; 10 μm **e**, **i**; 500 nm **j**–**l**

The autophagy–lysosome and ubiquitin–proteasome systems are the two major pathways that cells employ to selectively target long-lived and misfolded proteins, protein aggregates, damaged mitochondria, or unwanted cytoplasmic components and organelles [22]. We observed large autophagic vacuoles in the majority of cells during early reprogramming (Fig. 1a). In young passages of stable iPSCs, autophagic vacuoles were abundantly present but reduced in size (Fig. 1b). However, large autophagic vacuoles were absent in parental fibroblasts (Fig. 1f). TEM revealed the presence of lysosomal structures (Fig. 1e, i) in iPSCs. Structures of endosomal/lysosomal nature with double membranes were clearly evident under high magnification, which were filled with an array of cell debris and organelles (Fig. 1j–l).

Double immunofluorescence staining was carried out with anti-LC3B (an autophagy marker) and anti-LAMP1 (a major component of lysosomal membrane). In fibroblasts, fine dots of LC3B staining (Fig. 1g) were observed and most of them were not colocalized with LAMP1 (Fig. 1g). Similar to fibroblasts, small dots of LC3B staining were observed in iPSC culture which were not colocalized with LAMP1. However, strong LC3B staining appeared in large vacuoles (Fig. 1c) which colocalized with anti-LAMP1 staining, which could represent different status of LC3B activity. The control double staining was performed with anti-Syntaxin 6, a marker for Golgi membrane, and MitoGreen for mitochondria (Fig. 1d, h). As anticipated, there was no colocalization in either iPSCs (Fig. 1d) or fibroblasts (Fig. 1h). These data therefore demonstrate that large autophagic vacuoles are a characteristic feature of iPSCs which harbor both LC3B-II and LAMP1.

### iPSCs exhibit higher abundance of LC3B-II than parental fibroblasts

**Fig. 2:**
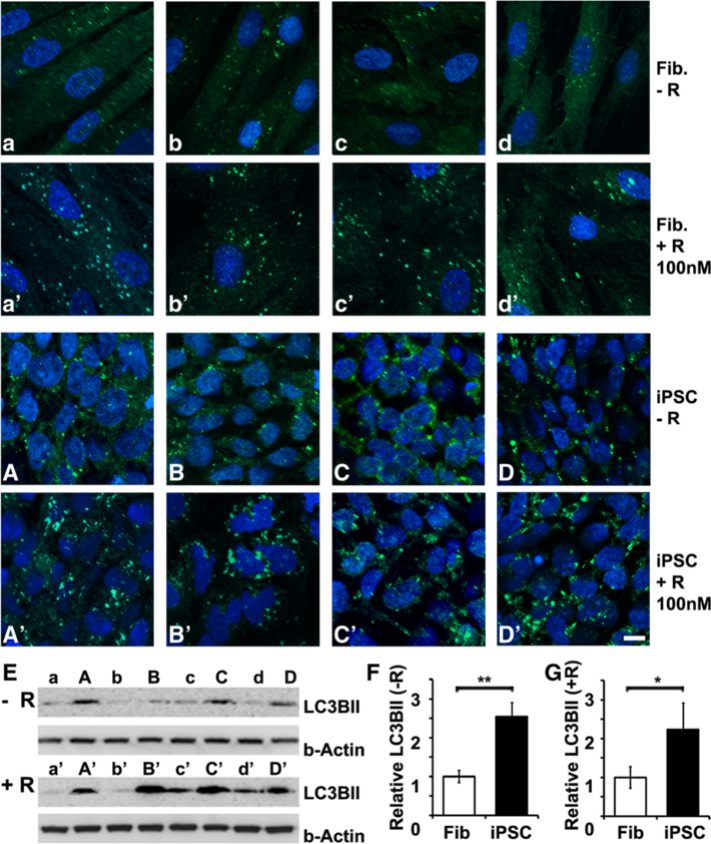
iPSCs exhibit higher autophagy activity than parental fibroblasts. Fibroblasts (**a**–**d**, **a**′–**d**′) and iPSCs (**A**–**D**, **A**′–**D**′) derived from four independent donors were cultured in the absence (*–R*) or presence (*+R*) of 100 nM rapamycin for 24 hours. iPSCs showed higher basal staining of LC3B (**A**–**D**) than in parental fibroblasts (**a**–**d**). LC3B was highly induced by 100 nM of rapamycin in iPSCs (**A**′–**D**′), with lower levels of induction in their respective fibroblasts (**a**′–**d**′). Fluorescent images were acquired via confocal microscopy. *Bar* = 10 μm. (**E**) Immunoblots of the protein extracts from untreated (–R) or treated (+R) cells with anti-LC3B-II and anti-β-ACTIN. (**F**, **G**) The relative abundance of LC3B-II was quantified using ImageJ software and data were presented as mean ± SD. (**F**) The basal level of LC3B-II in the iPSCs was 2.55-fold higher than in parental fibroblasts (**p < 0.01, *n* = 4). (**G**) Rapamycin-induced expression of LC3B-II in the iPSCs was also 2.23-fold higher than fibroblasts (*p < 0.05, *n* = 4). *Fib* fibroblasts, *iPSC* induced pluripotent stem cell

Next we compared iPSCs with parental fibroblasts from four independent donors by both immunocytochemistry (Fig. 2a–D′) and immunoblotting (Fig. 2E–G). The immunofluorescence study showed brighter basal LC3B-II staining in iPSCs (Fig. 2A–D) than in parental fibroblasts (Fig. 2a–d). Rapamycin (100 nM) induced autophagy in both iPSCs (Fig. 2A′–D′) and fibroblasts (Fig. 2a′–d′). Quantification of the immunoblots (Fig. 2E) showed 2.55-fold higher basal LC3B-II in the iPSCs than that in parental fibroblasts (Fig. 2F, *p* < 0.01, *n* = 4). After 1 day of rapamycin induction at 100 nM, LC3B-II was also 2.23-fold higher in the iPSCs than in fibroblasts (Fig. 2g, *p* < 0.05, *n* = 4). Overall, these data show that autophagy is highly active in human iPSCs and is rapamycin-inducible. Both the basal and induced autophagy is significantly higher in iPSCs than in dermal fibroblasts.

### High basal levels of autophagy components are expressed in iPSCs

**Fig. 3:**
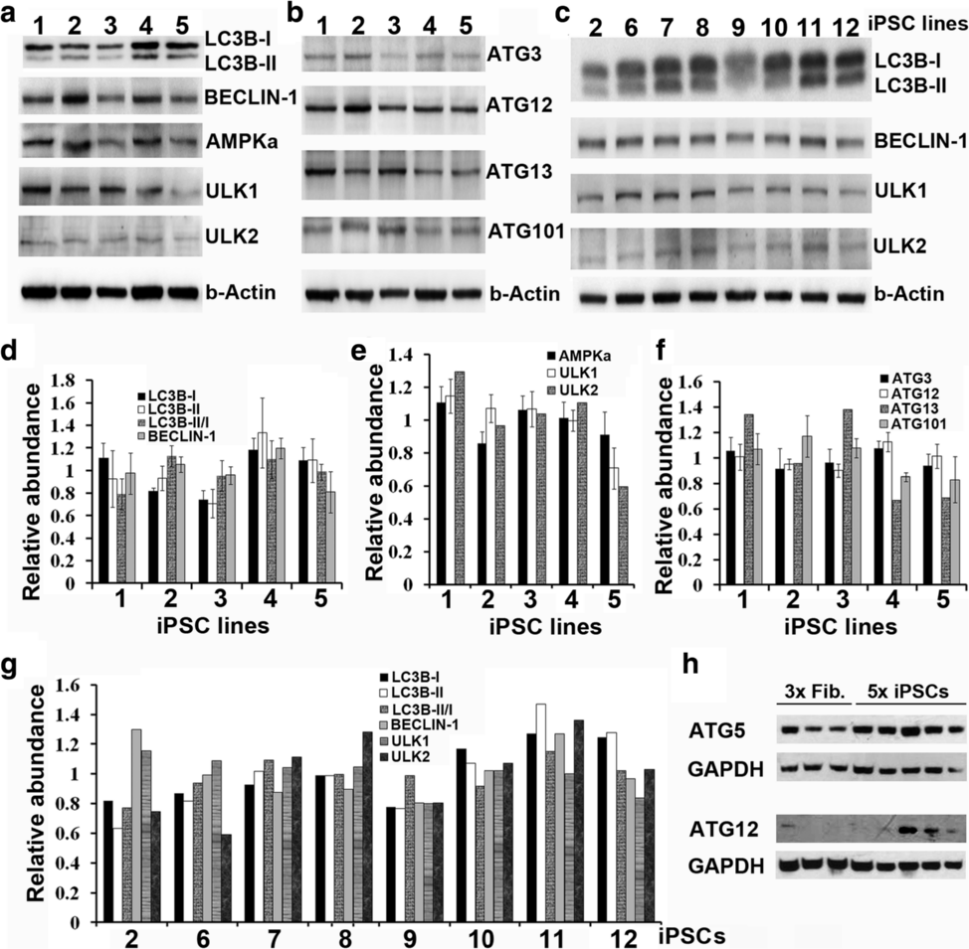
Wide expression of different autophagy components in independent iPSC lines. Proteins were extracted from iPSCs with daily renewal of culture medium. Then 15 μg of protein was loaded onto each lane. Lanes *a–l* represent 12 independent iPSC lines from 10 donors (1, 33D6; 2, JOM; 3, LV1; 4, LV2; 5, LV3; 6, 001CC1; 7, NRXN1C1; 8, 002 V; 9, 003 V; 10, SC126; 11, SC128; 12, SC132). (**a**–**c**) Immunoblotting was carried out with antibodies against LC3B-I, LC3B-II, BECLIN-1, AMPKα, ULK1, ULK2, ATG3, ATG12, ATG13, ATG101, and β-actin. (**d**–**g**) The relative abundance of the proteins was quantified using ImageJ software against β-actin and data were presented as mean ± SD. (**h**) Immunoblots were carried out to compare expression of ATG5 and ATG12 among three fibroblast lines (*3× Fib*.) and five iPSC lines (*5× iPSCs*) of healthy donors, showing higher ATG5 and ATG12 expression in iPSCs than that in fibroblasts. *iPSC* induced pluripotent stem cell

To further address the autophagy activity during iPSC maintenance, we determined basal expression levels of 10 autophagy members involving different steps of autophagy. Autophagy is repressed by the mTOR and activated by rapamycin. ULK1/2 are activated in a ULK1/2–Atg13/101–FIP200 complex [23, 24], which subsequently activates PI3K CIII complex (consisting of BECLIN-1, AMBRA, VPS34/15, and ATG14) and stimulates phagophore formation. ATG12 then conjugates with ATG5/16 and forms phagophores [25]. ATG4/7/3 then converts LC3B-I to LC3B-II to form autophagic vacuoles [17, 22, 26, 27]. We extracted proteins from 12 iPSC lines derived from 10 independent donors (Fig. 3), and carried out immunoblotting with antibodies against AMPKα, ULK1, ULK2, ATG13, ATG101, BECLIN-1, ATG3, ATG5, ATG12, and LC3B. Relative protein abundance was quantified against housekeeping proteins. AMPKα, BECLIN-1 ATG12, ATG13, and ULK1 were shown to be highly expressed in iPSCs, whereas ATG3, ATG101, and ULK2 were less abundant. No significant difference was detected among different lines for each component, but high levels of LC3B-II were detected in all iPSCs line (Fig. 3a, c). To further evaluate the difference between iPSCs and fibroblasts, we investigated ATG5 and ATG12 expression among three fibroblast lines and five iPSC lines. The iPSCs were consistently shown to have much higher ATG5/ATG12 expression compared with fibroblasts (Fig. 3h). These data demonstrate that most autophagy components are abundantly expressed in iPSCs.

### Rapamycin induces iPSC autophagy in concentration-dependent and time-dependent manners

To determine whether iPSC maintenance might benefit from upregulated autophagy, we investigated the dosage effect of rapamycin on phosphorylated ULK1, p70S6K, and the autophagy indicator—ratio of LC3B-II/I. Both ULK1 and p70S6K are serine/threonine kinases and targets of mTOR. p70S6K is a major regulator of translation and phosphorylated by mTOR [28–30], whereas ULK1 is an initiator of autophagy.

**Fig. 4:**
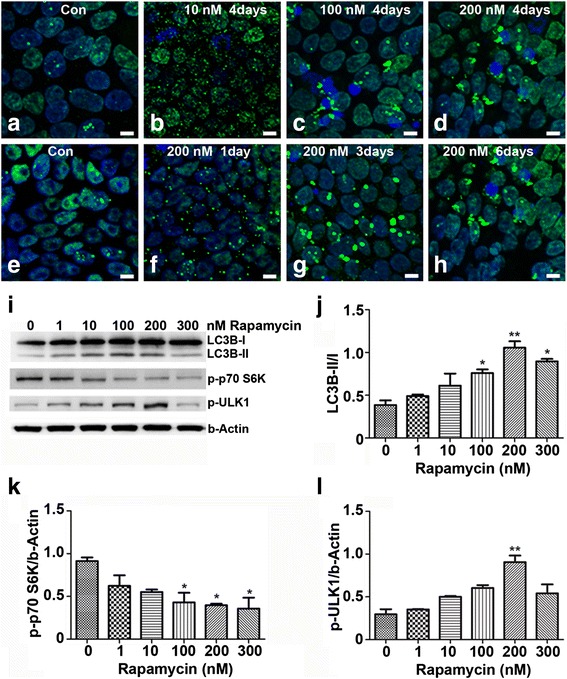
Rapamycin induces autophagy in time-dependent and concentration-dependent manners in iPSCs. iPSCs were maintained in six-well plates and medium was renewed daily. Images of LC3B staining were taken at 4 days of rapamycin treatment at 0, 10, 100, or 200 nM respectively (**a**–**d**) and for 200 nM of rapamycin treatment at 1, 3, and 6 days respectively (**e**–**h**), showing concentration-dependent and time-dependent induction. (**i**) Proteins were extracted from iPSCs treated with rapamycin at 0, 1, 10, 100, 200, or 300 nM for 4 days, and immunoblotted with antibodies against LC3B, phosphorylated ULK1, phosphorylated p70S6K, or β-actin. (**j**–**l**) Relative abundance of the LC3B-II/I ratios (**j**), p-p70S6K (**k**), and p-ULK1 (**l**) was quantified using ImageJ software against a loading control β-actin. Data were presented as mean ± SEM, *p < 0.05, **p <0.01. *Bar* = 10 μm **a**–**h**

We treated iPSCs with 0, 1, 10, 100, 200, or 300 nM of rapamycin for 4 days, and observed a dose-dependent reduction in p70S6K phosphorylation (Fig. 4i). The most significant reduction was achieved with 100–300 nM (Fig. 4k). In contrast, LC3B-II (Fig. 4j) and p-ULK1 (Fig. 4l) were enhanced in a dose-dependent manner, and the maximal increase was observed at 200 nM of rapamycin (*p* < 0.01). Similarly, immunostaining detected higher levels of LC3B-II expression in 200 nM rapamycin-induced cells (Fig. 4a–d).

We then treated iPSCs with 200 nM of rapamycin for 1, 3, and 6 days, observed significantly clustered LC3B-II staining after 3 days of rapamycin treatment, and showed a time-course-dependent activation of autophagy (Fig. 4e–h). This result was different from that for fibroblasts, in which the total LC3B was increased by 24 hours of rapamycin treatment whereas the LC3B-II remained relatively constant (Additional file 1: Figure S1). These data demonstrate that autophagy can be markedly activated by rapamycin in iPSCs.

### Rapamycin attenuates bafilomycin-induced iPSC death

**Fig. 5:**
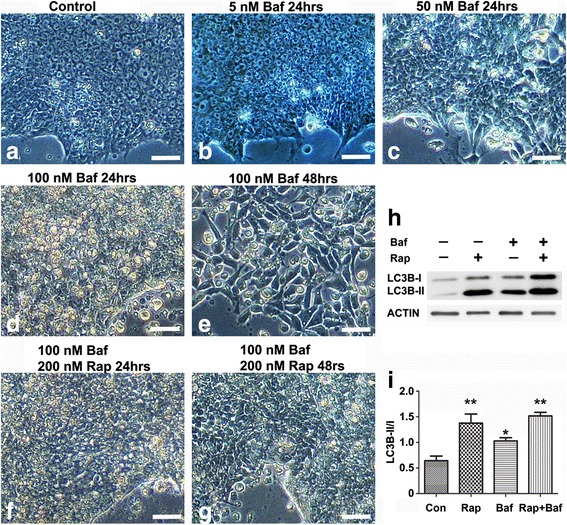
Rapamycin attenuates bafilomycin A1-induced iPSC death. iPSCs were treated with 0 (**a**), 5 nM (**b**), 50 nM (**c**), and 100 nM (**d**, **e**) of bafilomycin for 24 hours (**a**–**d**) or 48 hours (**e**). Bafilomycin A1 induced cell death in a concentration-dependent manner (**a**–**d**), leading to substantial cell loss after 48 hours at 100 nM (**d**). Addition of 200 nM rapamycin attenuated bafilomycin A1-induced cell death and maintained iPSC normal cell density (**f**, **g**). (**h**) Anti-ACTIN and Anti-LC3B immunoblotting were carried out with iPSC lysates from control iPSCs (*Con*), or 24 hours of treatment with 200 nm of rapamycin (*Rap*), or with 100 nM of bafilomycin A1 (*Baf*), or with both (*Rap + Baf*). (**i**) Relative abundance of the LC3B-II/I ratios was quantified against a loading control β-actin. Data were presented as mean ± SEM, *p < 0.05, **p <0.01, *n* = 4. *Bar* = 50 μm **a**, **b**

Bafilomycin A1 is an inhibitor of lysosome degradation by blocking the final stage of autophagy, fusion of autophagosomes with lysosome [31, 32]. We treated iPSCs with 0, 5, 50, and 100 nM of bafilomycin A1 for 24 or 48 hours, with or without 200 nM rapamycin. Bafilomycin A1 was shown to induce iPSC death in concentration/time-dependent manners. iPSC morphology was significantly altered after 24 hours of bafilomycin treatment at 50 nM (Fig. 5d, e) but not at 5 nM (Fig. 5b). At 100 nM, bafilomycin markedly induced iPSC death in 24 hours, leading to grossly reduced cell density after 48 hours (Fig. 5e). Addition of 200 nM rapamycin significantly attenuated bafilomycin-induced cell death, improved iPSC survival, and increased iPSC cell density at 48 hours of bafilomycin treatment (Fig. 5g).

Western blotting showed that bafilomycin enhanced both LC3B-I and LC3B-II bands by blocking basal autophagic flux. Rapamycin induced LC3B expression and caused a shift from LC3B-I to LC3B-II (Fig. 5h, i). Treatment of iPSCs with both rapamycin and bafilomycin A1 increased conversion of LC3B-II from LC3B-I similar to rapamycin treatment alone, thereby attenuating bafilomycin-induced cell death (Fig. 5c, d). These data further strengthen the importance of autophagy in iPSCs, because blockage of fusion of autophagosomes with lysosome rapidly induces iPSC death. Meanwhile, addition of rapamycin significantly improves iPSC survival.

### Prolonged treatment with rapamycin induces formation of uniform aggregates

**Fig. 6:**
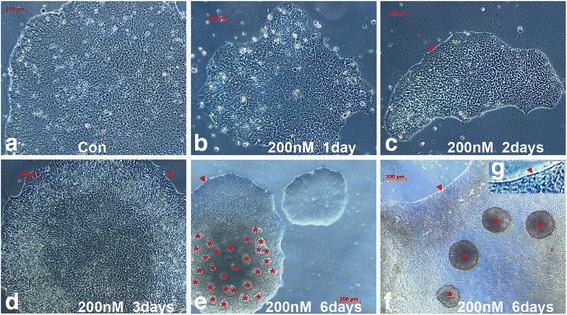
Time course of rapamycin-induced cell detachment and EB formation. iPSCs were cultured in the presence of 200 nM rapamycin. Bright-field microscopic images were taken as untreated control (**a**) and after 1 day (**b**), 2 days (**c**), 3 days (**d**) and 6 days (**e**, **f**) of treatment. EB-like spheres were spontaneously formed after 6 days of rapamycin treatment. (**c**, **e**, **f**) The border of the iPSC colonies formed a line after day 6 to limit colony expansion sideways (*arrowheads*). (**e**) Areas of iPSC detachment were filled with a thin layer of iPSCs (*). (**f**) Four EB-like spheres of comparable size were captured in the fields (*arrowheads*). (**g**) Magnified view (4×) of the border area with arrowheads in **f**. *Bar* = 100 μm **a**–**d**; 200 μm **e**, **f**

We next cultured iPSCs in Geltrex-coated dishes in the presence of 200 nM of rapamycin and examined the long-term effects on the maintenance of iPSCs. We first noticed the following morphological changes: the peripheral cells of the iPSC colonies first started to elongate (Fig. 6d) after 3 days, and cells at the edge produced an unknown matrix which formed a firm line at the border of the colony after 6 days to limit iPSC colony expansion sideway (Fig. 6f, g); rapamycin altered iPSC cell junctions, and cell borders became more obvious after 2 days of treatment (Fig. 6c, d); and iPSCs continued to proliferate in the central areas of colonies, together with the limitation to expand sideways and reduce cell–cell contacts, and round EB-like spheres were formed and detached from the culture dish before and after 6 days of rapamycin treatment (Fig. 6e, f).

**Fig. 7:**
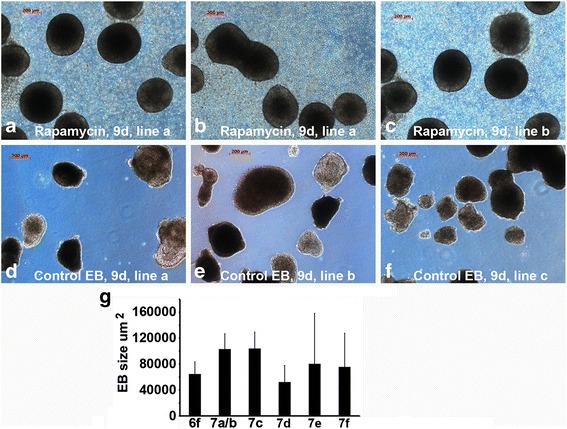
Rapamycin induces spontaneous formation of uniform sizes of EBs. (**a**–**c**) Images of floating spheres appeared after 9 days of rapamycin treatment (200 nM) from three iPSC lines. (**d**–**f**) Images of EBs made after 9 days from the cut-and-paste method. (**g**) EB area sizes (μm^2^) were quantified with ImageJ software and data were presented as mean ± SD. The X-axis in panel (**g**): 6f, 7a/b, 7c, 7d, 7e, 7f represents figure number, from which the EB sizes were quantified. Note the round shape and uniform sizes of EBs from rapamycin treatment (**a**–**c**), in contrast to irregular shape and sizes of EBs resulted from the same iPSC lines by mechanical method (**d**–**f**). *Bar* = 200 μm. *EB* embryoid body

The average aggregate size at 6 days was 64,793 ± 18,779 μm^2^ (*n* = 13). The round spheres continued to detach and grow after 9 days of treatment, and reached sizes of 112,251 ± 17,422 (002 V, *n* = 8), 104,640 ± 27,265 (JOM, *n* = 13), and 105,381 ± 13,468 μm^2^ (LV1, *n* = 7) in three independent iPSC lines, respectively. However, the control EBs resulting from the cut-and-paste method varied significantly in shape and size (Fig. 7f–h). These were 52,328 ± 25,518 (002 V, *n* = 10), 80,518 ± 77,708 (JOM, *n* = 20), and 75,652 ± 51,944 μm^2^ (LV1, *n* = 22), respectively, and displayed consistently large variation (SD) in sizes within each line (Fig. 7b). These data show that high concentrations of rapamycin treatment can induce iPSC detachment and spontaneous formation of uniformly sized aggregates.

### Rapamycin treatment accelerates differentiation of human iPSCs

**Fig. 8:**
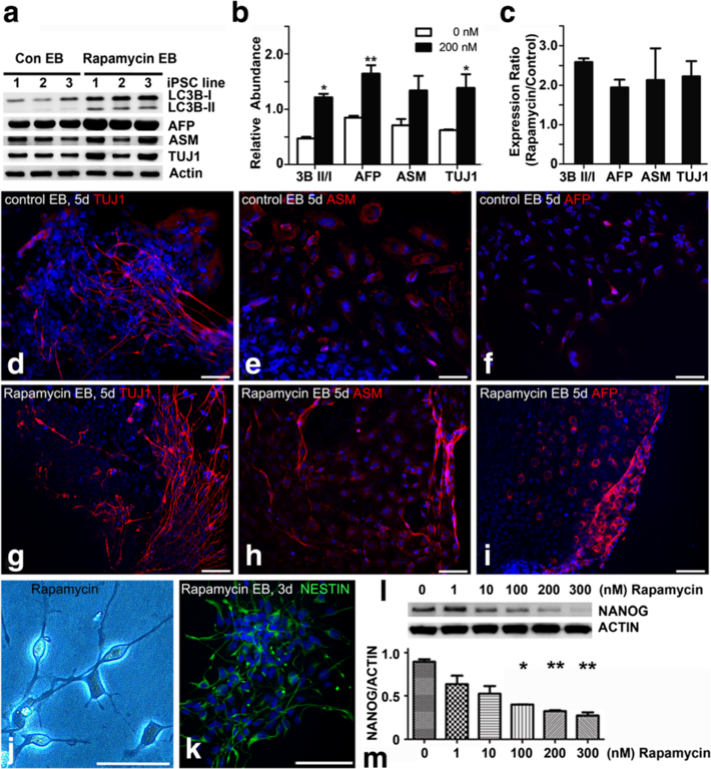
Rapamycin treatment accelerates differentiation of human iPSCs in association with decreased NANOG expression. **a** Proteins were extracted from rapamycin-induced spheres and control EBs, and blotted with antibodies against LC3B, AFP, ASM, TUJ1, and β-actin. 1, 2, 3 in panel (**a**) indicate source of proteins from three independent iPSC lines. **b** Relative protein expression was quantified against β-actin and (**c**) compared between two methods, presented as mean ± SEM. Note a 2–3-fold increase of LC3B, AFP, ASM, and TUJ1 expression in rapamycin-induced spheres. Rapamycin-induced spheres and control EBs were plated for spontaneous differentiation for 3 days (**j**, **k**) or 5 days (**d**–**i**), and stained for NESTIN (**k**), TUJ1 (**d**, **g**), ASM (**e**, **h**), or AFP (**f**, **i**). Note the presence of more immune-positive cells in derivatives of rapamycin-induced spheres (**g**–**i**) compared with cells differentiated from conventional EBs (**d**, **f**). (**l**) Proteins were extracted from iPSCs treated with rapamycin at 0, 1, 10, 100, 200, or 300 nM for 4 days, and immunoblotted with anti-NANOG, showing concentration-dependent reduction of NANOG expression after rapamycin treatment. **m** Relative abundance of NANOG expression after 4 days of rapamycin treatment. *Bar* = 100 μm **d**–**i**; 50 μm **j**, **k**. *p < 0.05, **p < 0.01. *Con* control, *EB* embryoid body

To determine the nature of the floating spheres resulting from rapamycin treatment, we extracted proteins from the aggregates and compared them with EBs generated via the cut-and-paste method by immunoblotting with three germ layer markers of α-fetoprotein (AFP for endoderm), α-smooth muscle actin (ASM for mesoderm), and βIII tubulin (TUJ1 for ectoderm), together with autophagy marker LC3B-II (Fig. 8a). The expression of these marker proteins was 2–3-fold higher in the rapamycin-induced spheres (Fig. 8a–c).

To examine the differentiation potential of aggregates, we plated them for spontaneous differentiation and comparison with EBs made mechanically. Cells with neuronal morphology appeared after 3 days without neuronal induction (Fig. 8j–l), and many were stained positive for neural stem cell marker NESTIN (Fig. 8m). After 5 days of spontaneous differentiation, we carried out immunocytochemical staining with AFP, ASM, and TUJ1 and obtained significantly higher proportions of cells positive for these makers in cells derived from rapamycin-induced spheres (Fig. 8g–i), compared with cells differentiated from the conventional EBs (Fig. d–f). To illustrate mechanism(s) of rapamycin-induced iPSC differentiation, we investigated NANOG expression after 4 days of treatment by immunoblotting (Fig. 8l). Rapamycin was shown to downregulate NANOG expression in a concentration-dependent manner, and significant reductions were detected at 100 nM (*p* < 0.05), 200 nM (*p* < 0.01), or 300 nM of rapamycin treatment (*p* < 0.01, Fig. 8m). These data suggest that rapamycin-induced spheres are similar to conventional EBs in pluripotency of differentiation, they are non-biased in differentiating into three lineages, but differentiate in an accelerated pace, in association with downregulated NANOG expression during EB formation.

### Rapamycin regulates adherens junctions and actin cytoskeleton in human iPSCs

**Fig. 9:**
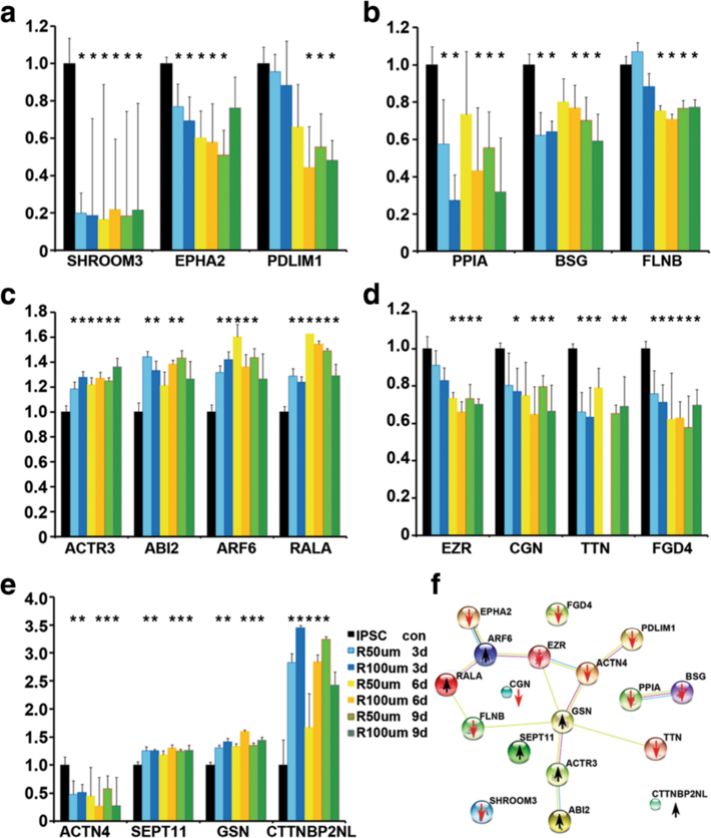
Rapamycin regulates adherens junctions and actin cytoskeleton pathways in iPSCs. Three independent lines of iPSCs were maintained in mTeSR™ (*con*) or in the presence of 50 or 100 nm rapamycin for 3, 6, or 9 days. Proteins were harvested for mass spectrometry analyses. (**a**–**e**) Time-course changes of protein expression (mean ± SEM, *n* = 3). *p < 0.05. (**f**) Molecular network for selected proteins whose expression was altered. Data show the network of interactions between adherens junctions and actin signaling by the STRING database. *Arrows* indicate upregulation or downregulation of the protein expression after rapamycin treatment. Each * represents *p* < 0.05 for the respective culture condition in comparison to the control iPSCs in black bar

To identify potential molecular mechanisms associated with rapamycin-induced iPSC detachment, we performed quantitative mass spectrometry analysis with protein extracts from control iPSCs and iPSCs treated with 50 or 100 nM of rapamycin for 3, 6, and 9 days respectively (*n* = 3 for each). A total of 6145 proteins were quantitatively identified, and 220 proteins were identified by statistical analyses, which were systematically altered at different time points and at both rapamycin concentrations (one-way ANOVA, two-tailed, *p* < 0.05). Gene ontology (GO) analyses of the 220 targets with the STRING database revealed that adherens junctions (17 hits, *p* = 6.49 × 10–^6^) and actin cytoskeleton (9 hits, *p* = 0.022) were significantly enriched in the rapamycin-treated cells (Fig. 9), which integrated into an interactive network (Fig. 9f).

Of the 17 adherens junction molecules, nine (ACTN4, BSG, EPHA2, EZR, FLNB, PDLIM1, PPIA, SHROOM3, RPLP0) were downregulated and eight (ABI2, GSN, ACTR3, ARF6, RALA, RPL7A, RPL19, RPL27) were upregulated (Fig. 9a–c). Of the nine actin cytoskeleton-associated proteins, six (ACTN4, CGN, EZR, FGD4, SERPINA3, TTN) were downregulated and three (CTTNBP2NL, GSN, SEPT11) were upregulated (Fig. 9d, e). Consistent with the morphological changes, these data suggest that alterations of adherens junctions and cytoskeleton are the key pathways in rapamycin-induced iPSC behavior.

## Discussion

Autophagy is a cellular mechanism to maintain minimal cell activity for viability in response to nutrient limitations and cell stress, by degrading and recycling cytoplasmic proteins and subcellular organelles via the fusion of a double-membrane-bound vesicle, autophagosome, and lysosome [17, 33]. Autophagy plays an essential role in cellular reprogramming and the basal level of autophagy increases in aging human skin fibroblasts, which may affect different reprogramming efficiency [34]. There appears no correlation with sex (all male) or age (4–20 years) in the fibroblast lines we have used, but there is correlation with heath status, which requires validation in larger sample sets. In this study we demonstrate abundant presence of large autophagic vacuoles in early fibroblast reprogramming and smaller ones during iPSC maintenance. We show a high basal level of key components of autophagy in 12 iPSC lines derived from 10 independent donors, and prolonged rapamycin treatment resulted spontaneous formation of uniformed sized EBs with accelerated differentiation pace.

In previous studies, lack of autophagy components of Atg3, Atg5, or Atg7 was shown to render MEF cells defective in iPSC colony formation [15], and lack of two different autophagy genes, *atg5* and *beclin1*, displayed a defect in EB formation during development [35]. The reprogramming was entirely blocked also in *Tsc2* ^–/–^ somatic cells with hyperactivated mTOR activity which suppressed autophagy [14]. Subtle tuning of the mTOR activity with inhibitors (i.e., 0.3–1 nM rapamycin or 0.1 nM PP242) was found to increase reprogramming efficiency [18]. Mechanistically, the reprogramming factor Sox2 could bind to mTOR promoter and repress mTOR expression and activate autophagy [15]. These data together showed that reduced mTOR activity and elevated autophagy were required during cellular reprogramming, which is consistent with our observation that abundant large autophagic vacuoles are present in early fibroblast reprogramming, which become smaller during the subsequent passaging.

Balanced autophagy and mTOR activity is also essential for the maintenance and differentiation of pluripotent stem cells. Human ES cells were shown previously to have a tight regulation of the mTOR signaling to mediate protein translation for maintaining the pluripotent status [36]. Activation of p70S6K, a mTOR downstream factor, was shown to induce differentiation of human ES cells [36], whereas inhibition of mTOR autophosphorylation by 20 nM rapamycin was reported to disrupt p70S6K-mediated translation, but not to alter cell viability or expression of the pluripotency markers [37]. In contrast, the mTOR was necessary for growth and proliferation of early mouse embryos and ES cells, and disruption of the mouse mTOR gene prohibited ES cell development [38, 39]. Interestingly, 100 nM rapamycin was previously shown to reduce the size and rate of EB formation in human amniotic fluid stem cells [40]. In contrast, our study demonstrated that a high concentration of rapamycin induced EB formation in iPSCs. Inhibition of the mTOR activity with high concentrations of rapamycin also greatly impaired somatic cell reprogramming [14], and 100 nM rapamycin was used to primer hepatocyte differentiation of iPSCs prior to Activin A induction [41]. This is consistent with our data that rapamycin downregulates NANOG expression in a concentration-dependent manner and accelerates iPSC differentiation.

To uncover rapamycin pathways in iPSCs, we carried out quantitative mass spectrometry. The STRING Gene Ontology analyses revealed that actin cytoskeleton and adherens junctions were the integrated pathways regulated by rapamycin. For example, ACTN4, BSG, CGN, EPHA2, EZR, FGD4, FLNB, PDLIM1, PPIA, SHROOM3, RPLP0, SERPINA3, and TTN were significantly downregulated, whereas ABI2, CTTNBP2NL, GSN, ACTR3, ARF6, RALA, RPL7A, RPL19, RPL27, and SEPT11 were upregulated.

Actin is critical for cell shape, adhesion, and migration. The dynamics of actin cytoskeleton are modulated by rapamycin targets of SHROOM3, EZR, and GSN. For example, SHROOM3 is required for the apical localization of F-actin/myosin II [42]. EZR links the plasma membrane to actin and is involved in adhesion and migration. Inhibition of EZR expression reduced adhesiveness in colorectal cancer cells [43]. GSN knockdown decreased cell viability and tumor cell invasion, whereas GSN overexpression correlates with proliferative and invasive capacities [44]. BSG promotes cell–cell adhesion, and its downregulation altered the actin/Spectrin network [45]. Reduced expression of SHROOM3, EZR, and BSG in the rapamycin-treated iPSCs is therefore in line with reduced cell adhesion in the literature.

ACTN4, FLNB, and PDLIM1 are actin-binding proteins. ACTN4 is concentrated at sharp extension and at the edge of cell clusters [46], and FLNB regulates direct communication between the cell membrane and the cytoskeletal network [47]. PDLIM1 functions as an adapter to recruit other LIM-interacting proteins to the cytoskeleton, and suppression of PDLIM1 resulted in cell spreading and the loss of stress fibers and focal adhesions [48]. In addition, ACTR3 is also essential to cell shape and motility, and is an ATP-binding component of the ARP2/3 complex, which regulates actin polymerization. The complex can be activated by ABI, and knockdown of ABI markedly inhibits cell–cell junctions [49]. Rapamycin-induced changes in the expression of ACTN4, ACTR3, FLNB, and PDLIM1 are therefore also likely to affect actin cytoskeleton.

Some of the rapamycin targets are GTPase activity related, such as downregulated EPHA2 and upregulated ARF6 and RALA. GTPases are involved in cell adhesion migration and oncogenic transformation. For example, EphA2 is transmembrane receptor tyrosine kinase activating and prompts cells to round up and detach from their neighbors [50]. RALA is a small GTPase, and constitutively active RALA promotes anchorage-independent growth signaling [51]. ARF6 is a small GTPase to balance with RAB35 GTPase by cells during cell migration and adhesion. Increased ARF6 activity from RAB35 knockdown enhances cadherins accumulation and reduces cell–cell adhesion. The loss of RAB35, however, correlates with enhanced cell migration [52]. Reduced EPHA2 and increased ARF6 and RALA expression may therefore also contribute to rapamycin-induced iPSC detachment. Together these data showed that rapamycin regulates an array of adherens junctions and actin-modifying molecules, which collectively alter iPSC adhesion.

## Conclusions

In this study, we demonstrate that autophagy is a prominent feature of reprogramming cells and stable iPSCs. The basal level of autophagy is universally present among human iPSC lines, which is significantly higher than parental fibroblasts. Block of autophagy by bafilomycin induces iPSC death. Rapamycin activates autophagy in concentration-dependent and time-dependent manners and attenuate bafilomycin toxicity in iPSCs. Prolonged rapamycin treatment induces cell detachment and formation of uniformly sized EBs from human iPSCs, with altered expression of adherens junctions and actin-modifying molecules. Rapamycin downregulates NANOG expression, induces EB formation, and accelerates cell differentiation into three germ-layer lineages. These findings are significant in two ways. Firstly, the 3D microenvironment including shape and size of EBs can affect differentiation. The iPSCs are supersensitive to enzyme dissociation and therefore the “hanging drop” technique is difficult to apply, despite being available to generate uniform EBs from ES cells over 20 years ago. Rapamycin may therefore assist generation of uniformly sized EBs with round shape, which may reduce the heterogeneity of the end cell types. Secondly, differentiation of mature and functional cell types from human iPSCs is time consuming, and the accelerated differentiation associated with rapamycin treatment is promising in shortening the differentiation duration. Rapamycin may therefore help overcoming the challenges associated with phenotypic characterization, drug discovery, and cell replacement therapy for neurological disorders.

## Additional file

Additional file 1: Figure S1. Showing that rapamycin induces LC3B expression in human fibroblasts. Three lines of human fibroblasts (*F1*, *F2*, *F3*) were treated without (*–*) or with (*+*) 100 nM rapamycin overnight. The protein lysates were run of 15 % SDS-PAGE and blotted with anti-LC3B and anti-GAPDH. Two bands of LC3B-I (16 kDa) and LC3B-II (14 kDa) were seen. Rapamycin is shown to induce LC3B-I expression after 24 hours of treatment. (TIF 172 kb)

## Abbreviations

AFP: α-Fetoprotein
ANOVA: Analysis of variance
ASM: α-Smooth muscle actin
*ATG*: Autophagy-related gene
EB: Embryoid body
ES: Embryonic stem
iPSC: Induced pluripotent stem cell
LC3I: Microtubule-associated proteins 1A/1B light chain 3-I
MEF: Mouse embryonic fibroblasts
mTOR: Mammalian target of the Rapamycin
NaB: Sodium butyrate
PE: Phosphatidylethanolamine
PVDF: Polyvinylidene difluoride
SEM: Standard error of means
TEM: Transmission electron microscopy
TUJ1: βIII tubulin
