# Supplementary figures and images for: Rapamycin regulates autophagy and cell adhesion in induced pluripotent stem cells
Source: Stem Cell Res Ther. 2016 Nov 15;7:166. doi: 10.1186/s13287-016-0425-x (PMC5109678; doi:10.1186/s13287-016-0425-x)

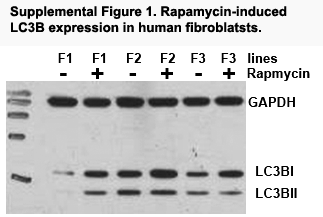

Supplement: Additional file 1: Figure S1. — Showing that rapamycin induces LC3B expression in human fibroblasts. Three lines of human fibroblasts (F1, F2, F3) were treated without (–) or with (+) 100 nM rapamycin overnight. The protein lysates were run of 15 % SDS-PAGE and blotted with anti-LC3B and anti-GAPDH. Two bands of LC3B-I (16 kDa) and LC3B-II (14 kDa) were seen. Rapamycin is shown to induce LC3B-I expression after 24 hours of treatment. (TIF 172 kb) [file 13287_2016_425_MOESM1_ESM.tif]
